# Relative Validity of Dietary Total Antioxidant Capacity for Predicting All-Cause Mortality in Comparison to Diet Quality Indexes in US Adults

**DOI:** 10.3390/nu12051210

**Published:** 2020-04-25

**Authors:** Kyungho Ha, Kijoon Kim, Junichi R. Sakaki, Ock K. Chun

**Affiliations:** 1 Department of Nutritional Sciences, University of Connecticut, Storrs, CT 06269, USA; kyungho.ha@uconn.edu (K.H.); drkijoon@gmail.com (K.K.); junichi.sakaki@uconn.edu (J.R.S.); 2 Department of Food and Nutrition, Sookmyung Women’s University, Seoul 04310, Korea

**Keywords:** antioxidant, total antioxidant capacity, diet quality index, mortality, U.S. adults

## Abstract

While traditionally diet quality index scores (DQIS) as noted later in this abstract have been used to predict health outcomes, dietary total antioxidant capacity (TAC), a useful tool for assessing total antioxidant power in the diet, may also be a novel predictor. This study evaluated the associations between dietary TAC and DQIS and all-cause mortality. Based on the National Health and Nutrition Examination Survey (NHANES) 1988–1994 and 1999–2006, 23,797 US adults were followed-up until 2015. Dietary TAC and DQIS including the Healthy Eating Index-2015 (HEI-2015), Alternative Healthy Eating Index-2010 (AHEI-2010), alternate Mediterranean Diet (aMED), and Dietary Approaches to Stop Hypertension (DASH) were calculated using a 1-day 24 h dietary recall. US adults in the highest quintiles of DQIS had lower rates of all-cause mortality compared to those in the lowest quintiles (HEI-2015 hazard ratio (HR): 0.87, 95% confidence interval (CI): 0.77–0.98; AHEI-2010 HR: 0.84, 95% CI: 0.74–0.94; aMED HR: 0.79, 95% CI: 0.69–0.90; DASH HR: 0.80, 95% CI: 0.70–0.92). Similarly, those in the highest quintile of dietary TAC also had a lower all-cause mortality than those in the lowest quintile (HR: 0.88, 95% CI: 0.79–0.98). These findings suggest that dietary TAC might be a relatively valid predictor of all-cause mortality in the US population compared to the DQIS.

## 1. Introduction

Poor diet quality is thought to be a leading risk factor for reduced quality of life as well as increased mortality [1]. To date, most dietary guidance to promote health is largely based on data about single foods and nutrients, failing to capture multiple dimensions of diet. Thus, several diet quality indexes have been developed based on the a priori approach to determine conceptually defined dietary components that are considered important for the promotion of health [2,3]. For example, Healthy Eating Index (HEI), Alternative Healthy Eating Index (AHEI), alternate Mediterranean Diet (aMED), and Dietary Approaches to Stop Hypertension (DASH) have been widely used to evaluate American diets [2]. These diet quality indexes have been used in epidemiologic studies to reflect risk gradients for major diet-related diseases [4]; however, the methods for measuring diet indexes are complicated and their relationships with disease risk vary across studies [5,6,7], making it difficult to formulate public health recommendations for a healthy diet and guidelines for self-assessment of diet quality. 

Oxidative stress indicates an imbalance between reactive oxygen species (ROS) production while an antioxidant defense system reflects the ability to counteract these products in cells and tissues [8,9,10]. Normally, the endogenous antioxidant defensive system in our body can regulate the ROS formation [8,11]. However, a high level of oxidative stress can lead to oxidative damage to cellular structures such as DNA, proteins, and lipids and contribute to pathogenesis and progression of several chronic diseases including cancer, cardiovascular diseases (CVDs), chronic obstructive pulmonary disease, and diabetes [8,10], which are leading causes of death in the US [12]. 

As radical scavengers, dietary antioxidants suppress ROS generation [8]. Antioxidants can be obtained externally and diets rich in fruits and vegetables play a crucial role in providing antioxidants such as vitamin C, vitamin E, carotenoids, including β-carotene, α-carotene, β-cryptoxanthin, lycopene, lutein, and zeaxanthin, and flavonoids. Considering that the usual diet consists of antioxidants in various chemical forms with different degrees of antioxidant capacities and that these combined antioxidants may exert cumulative or synergistic effects, dietary total antioxidant capacity (TAC) has received attention as a useful tool for assessing total antioxidant power in the diet [13,14,15,16]. However, the utility of dietary TAC has been debated due to the fact that estimation of dietary TAC varies widely by measurement methodology and that dietary TAC might not directly reflect plasma TAC [16]. Nevertheless, high dietary TAC has been consistently reported to be associated with lower biomarkers indicative of oxidative stress [17,18,19,20] and risk of chronic diseases such as CVDs [21,22,23,24,25], diabetes [26,27], and cancer [28,29,30], regardless of methodological disparities in measuring dietary TAC. Thus, dietary TAC may be a novel predictor of major health outcomes as they relate to oxidative stress and deserves investigation as a measure of diet quality considering its convenience and effectiveness in assessing antioxidant-rich dietary patterns.

To date, there is one previous study that examined associations between dietary TAC and several diet quality index scores (DQIS), and reported dietary TAC showed a positive correlation with DQIS [31]. However, it was conducted for small samples of healthy young adults and estimated TAC for limited food items. In addition, few studies have compared dietary TAC and diet quality indexes in the context of a relationship with health outcomes. Our research group developed a simple algorithm to theoretically calculate TAC by standardizing antioxidant intake data with the vitamin C equivalent antioxidant capacity of individual antioxidants [32]. This innovative protocol has been validated in human serum [33] and urine [34]. Dietary TAC estimated based on this protocol is further validated for its relevance, reliability, and predictability for in vivo antioxidant and/or oxidative stress status under different pathological clinical conditions [19,30,33,35,36,37,38] and for its association with all-cause and CVD mortality [39]. Therefore, using this algorithm, this study aimed to expand our study by assessing the relative predictive validity of dietary TAC, in comparison with key DQIS, for the reduction of the risk of all-cause mortality in a representative sample of the US population.

## 2. Materials and Methods 

### 2.1. Study Population

This study utilized data of US adults from the National Health and Nutrition Examination Survey (NHANES) III (1988–1994) and 1999–2006, which is an ongoing nationwide survey to evaluate the health and nutritional status of the non-institutionalized US population. The NHANES study protocols were approved by the National Centers for Health Statistics Research Ethics Review Board and documented consent was obtained from all participants. Detailed explanations on the NHANES are available elsewhere [40,41]. Participants were excluded for ineligible mortality status (n = 35), unreliable or incomplete dietary recall data (n = 4399), implausible total energy intake (<500 or >5000 kcal/day) (n = 608), history of cancer (n = 1680), pregnant or breastfeeding (n = 483), and dietary TAC of more than mean + 3SD (n = 485). Among the 31,487 eligible participants aged 30 years or older who completed a 1-day 24 h dietary recall, a total of 23,797 participants were included in the final analysis. The baseline characteristics of the study participants in the NHANES III and 1999–2006 are shown in Table S1.

### 2.2. Estimation of Dietary TAC 

Antioxidant intake was estimated using a 1-day 24 h dietary recall administered by a trained interviewer. The participants reported all foods and beverages that were consumed within a 24 h period. Antioxidant vitamins such as vitamin C, vitamin E (α-tocopherol), and carotenoids (β-carotene and α-carotene) were calculated from the dietary recall data. Other antioxidants including flavonoids, isoflavones, and proanthocyanidins were estimated by linking the dietary recall data to a flavonoid database which has been expanded by our research team using the latest USDA databases for flavonoids [42], proanthocyanidins [43], isoflavones [44], and the expanded flavonoid database [45]. The expansion protocol of the flavonoid database can be found elsewhere [46]. For each participant, daily antioxidant intakes were estimated by multiplying food intake and the contents of individual antioxidants for each food. Each antioxidant has a different antioxidant capacity and the antioxidant capacity of each individual antioxidant was measured using the 2,2′-azino-bis-3-ethylbenzthiazoline-6-sulphonic acid (ABTS) assay and expressed as vitamin C equivalents (VCE) [32]. To yield dietary TAC, daily antioxidant intake was multiplied by antioxidant capacity of each antioxidant (VCE) and then individual antioxidant capacities were summed up.

### 2.3. Estimation of Diet Quality Index Scores

To estimate DQIS, food group intakes were calculated using the Pyramid Servings Database (PSDB) developed by the National Cancer Institute and the MyPyramid Equivalents Database (MPED) and Food Patterns Equivalents Database (FPED) developed by the USDA. The PSDB was applied for the NHANES III and the MPED 1.0, and MPED 2.0 were applied for the NHANES 1999–2004. The FPED 2005–2006 was applied for the NHANES 2005–2006. Based on these databases, the number of servings from each food group was obtained. Since there was no information on sugar-sweetened beverage (SSB) and low-fat dairy intakes in these databases, we calculated SSB and low-fat dairy intakes using What We Eat in America Food Categories. Based on intakes of food groups and several nutrients, four different DQIS including HEI-2015, AHEI-2010, aMED, and DASH were calculated.

Firstly, the HEI-2015 was designed based on the 2015–2020 Dietary Guidelines for Americans and contains 9 components related to adequacy (total fruits, whole fruits, total vegetables including legumes, greens and beans (dark green vegetables and legumes), whole grains, dairy, total protein foods, seafood and plant proteins, and fatty acids) and 4 components related to moderation (refined grains, sodium, added sugars, and saturated fatty acids) [47]. These components were scored using a density basis (per 1000 kcal), except fatty acids, which is a ratio of unsaturated to saturated fatty acids, and each component has a standard for a minimum (0 points) or maximum (5 or 10 points) score. The scores for each component were summed to calculate a total score ranging from 0 to 100, with a higher score indicating better adherence to the 2015–2020 Dietary Guidelines.

Secondly, the AHEI-2010 is an alternative index to the HEI on the basis of scientific evidence of dietary factors related to the development of chronic disease [48]. The AHEI-2010 was originally composed of 11 components including whole fruit, vegetables except potatoes, whole grains, nuts and legumes, SSBs and fruit juice, red and processed meats, n-3 fats (EPA and DHA), polyunsaturated fat, trans fat, sodium, and alcohol; however, in this study, trans fat could not be considered due to the lack of trans fat intake in the dietary recall data. Similar to the HEI-2015, each component was scored from 0 points to 10 points and the total score of the AHEI-2010 ranged from 0 to 100, with a higher score indicating better adherence.

The aMED is an index to represent adherence to the Mediterranean diet for US population and includes total fruits, vegetables except potatoes, whole grains, legumes, nuts, fish, red and processed meats, ratio of monounsaturated to saturated fat, and alcohol [49]. The participants received 1 point if their intakes were greater than the median except red and processed meats and alcohol. Red and processed meat intake less than the median and alcohol intake of 10–25 g/day for men and 5–15 g/day for women received 1 point. If these criteria were not satisfied, 0 points were assigned. Thus, the total score of the aMED ranged from 0 to 9, with a higher score indicating greater adherence to the Mediterranean diet.

Lastly, the DASH score reflecting compliance to a DASH-style diet was calculated for 8 components, including total fruits, vegetables except potatoes, whole grains, nuts and legumes, low-fat dairy, red and processed meats, SSBs, and sodium [50]. Based on quintiles intake of total fruits, vegetables except potatoes, whole grains, nuts and legumes, and low-fat dairy, the highest quintile was assigned a score of 5 and the lowest quintile was assigned a score of 1. On the other hand, regarding red and processed meats, SSBs, and sodium, the lowest quintile was given 5 points and the highest quintile was given 1 point. By summing up the points for each component, the DASH score ranged from 8 to 40, with a higher score indicating greater adherence to the DASH-style diet.

### 2.4. Outcome

All-cause mortality was the outcome, and mortality data was obtained from the National Center for Health Statistics Linked Mortality Files [51]. The mortality status of the participants was ascertained by linking with the National Death Index and was followed up until 31 December 2015. Person-time was calculated from the date of interview to the date of death or the end of follow-up.

### 2.5. Statistical Analysis

All statistical analyses were conducted using SAS software (version 9.4; SAS Institute, Cary, NC, USA). Survey weights, cluster, and strata of the NHANES were applied using PROC SURVEY procedures. Continuous variables were expressed as mean ± SE and categorical variables were expressed as percentages. Dietary TAC was energy-adjusted using the residual method. The participants were divided into quintiles according to DQIS and dietary TAC. Differences in baseline characteristics according to DQIS and dietary TAC were examined using a general linear model for continuous variables and chi-square test for categorical variables. To evaluate associations between diet quality and dietary TAC, food and nutrient intakes and DQIS according to quintile of dietary TAC were estimated after adjusting for age (in 5-year categories), gender, race/ethnicity (white, black, Hispanic, or other), body mass index (BMI) (kg/m^2^, continuous), and total energy intake (kcal/day, continuous). For all continuous variables, log-transformed values were used for statistical tests to normalize distribution and a post-hoc Tukey-Kramer test was performed. Cox proportional hazard models were used to estimate hazard ratios (HRs) and 95% confidence interval (CIs) for all-cause mortality according to quintiles of DQIs and dietary TAC based on three models: Model 1 adjusting for age, gender, race/ethnicity, BMI, and total energy intake, Model 2 additionally adjusting for poverty-income ratio (<1.3, 1.3–<1.85, or ≥1.85) as an index of family income, marital status (married/unmarried), physical activity (sufficient/insufficient), history of cardiovascular diseases (yes/no), history of diabetes (yes/no), and history of hypertension (yes/no), and Model 3 additionally adjusting for smoking (never and former smokers who stopped smoking for ≥3 years, former smokers who stopped smoking for <3 years, or current smokers). The proportional hazard assumption was checked using log-log plots. Linear trends were estimated using the median value of each quintile and also estimated treating quintiles as continuous variables for comparison in Cox proportional hazard models only. All *p* values were two-sided and *p* < 0.05 was considered as statistically significant.

## 3. Results

Baseline characteristics according to quintiles of DQIS and dietary TAC are shown in Table 1. People in the highest quintiles of DQIS and dietary TAC were more likely to be women, older, and white and have higher family income levels compared to those in the lowest quintiles (*p* < 0.05 for all). The proportion of the participants who had sufficient physical activity level and who never smoked and smoked in the past but quit for ≥3 years was higher in the highest quintiles of DQIS and dietary TAC than in the lowest quintiles (*p* < 0.0001 for all).

All DQIS were positively associated with dietary TAC, although DQIS were highest in quintile 4 and slightly lower in quintile 5 than in quintile 4 (*p* for trend <0.0001 for all) (Table 2). Dietary TAC was also related to food group intakes, and intakes of total fruits including whole fruits and fruit juice, dark green vegetables, whole grains, legumes, and nuts and seeds showed increasing trends by dietary TAC (*p* for trend <0.05 for all). Total protein food intake was not significantly associated with dietary TAC. However, when divided into specific food groups, red and processed meat intake was inversely associated with dietary TAC (*p* for trend <0.0001), whereas seafood intake was positively associated with dietary TAC (*p* for trend = 0.0058). On a nutrient level, monounsaturated and saturated fatty acid intakes showed an inverse association with dietary TAC (*p* for trend <0.0001 for all).

During 14.9 years of follow-up, 8106 deaths were documented. Table 3 shows multivariable-adjusted HRs and 95% CIs for all-cause mortality according to quintiles of DQIS and dietary TAC. After adjusting for confounders in model 3, people in the highest quintiles of DQIS had lower rates of all-cause mortality compared to those in the lowest quintiles, although there was no significant decreasing trend with HEI-2015 (HEI-2015 HR: 0.87, 95% CI: 0.77–0.98, *p* for trend = 0.1178; AHEI-2010 HR: 0.84, 95% CI: 0.74–0.94, *p* for trend = 0.0082; aMED HR: 0.79, 95% CI: 0.69–0.90, *p* for trend <0.0001; DASH HR: 0.80, 95% CI: 0.70–0.92, *p* for trend = 0.0074). Dietary TAC showed a similar association to DQIS, with those in the highest quintile of dietary TAC also having a 12% lower rate of all-cause mortality than those in the lowest quintile (HR: 0.88, 95% CI: 0.79–0.98). However, a linear trend estimated using the median value of quintiles did not remain significant when smoking was additionally adjusted in the same model (*p* for trend = 0.2553), while a linear trend estimated using quintiles as continuous variables remained significant (*p* for trend = 0.0293).

## 4. Discussion

Based on a large sample of nationally representative US adults, the present study found that dietary TAC was positively associated with the DQIS including HEI-2015, AHEI-2010, aMED, and DASH and people in the highest quintile of dietary TAC had a lower rate of all-cause mortality than those in the lowest quintile, similar to the trends observed with DQIS.

The link between dietary TAC and mortality is still a rather novel concept with little research conducted, particularly in an American population. However, this study’s findings were in agreement with previous findings that dietary TAC was associated with reduced all-cause mortality among adults in Spain [52], Sweden [53], and France [54]. One study reported no significant association among Spanish adults [55], but this study was conducted with relatively few participants and a short follow-up period. Dietary TAC estimated by the same methodology of this study also showed inverse associations with CVD mortality [39], C-reactive protein level [20], and plasma total homocysteine level [56] in US adults, which have been reported as influential factors for mortality [57,58].

The diet quality index has been established in various ways in terms of theoretical framework, target group, target disease, scoring algorithm, and so on [2]. Adding the aforementioned information on four DQIS used in this study, these indexes were developed for US population and are related to reduce chronic disease risks except for DASH targeted mainly towards hypertension [47,48,49,50]. With regard to differences in these four indexes besides the dietary components, the HEI-2015 and AHEI-2010 are metric-scaled indicators based on normative cutoff values, whereas the DASH and aMED are ordinal (DASH) or dichotomous (aMED) indicators based on percentile cutoff values such as median and quintile values [2]. Nevertheless, all DQIS showed similar inverse associations with all-cause mortality in US adults, although the strengths of association differed slightly.

In general, US adults with high dietary TAC showed higher adherence to the DQIS. High consumption of fruits, vegetables except potatoes, whole grains, and nuts and legumes are commonly emphasized in all DQIS and this study found that dietary TAC was positively associated with these food intakes. These food groups have been consistently reported as major sources of TAC [20,59,60] and are also known as healthy plant foods [61,62]. Overall fatty acid intakes tended to decrease by dietary TAC due to low fat content of high TAC foods, but a decreasing trend of red and processed meat intake by dietary TAC was observed as all DQIS except HEI-2015 have encouraged. Meanwhile, HEI-2015 has recommended high consumption of seafood and legumes and the present study found that seafood intake was slightly increased by dietary TAC.

On the other hand, there was no significant linear trend in sodium and added sugar intakes according to dietary TAC, while participants in the highest quintile of dietary TAC consumed lower added sugars than those in the lowest quintile. Previous studies reported a positive association between sodium intake and dietary TAC among Korean adults [63] and French women [64], which might reflect their typical major sources of vegetable intakes such as Kimchi among Koreans. SSB consumption was highest in the lowest quintile of dietary TAC and the second highest in the highest quintile of dietary TAC in this study. This finding can be partly explained by the fact that tea, fruit juices, and other drinks are major sources of dietary TAC in US adults [59,60]. Considering that SSBs including fruit juices are attributable to high consumption of added sugars in US adults [65] and sodium and added sugars may induce endothelial dysfunction and CVDs mediated by oxidative stress [66,67], these nutrients should be carefully monitored when applying dietary TAC to the public health fields. Further epidemiological investigations on interactions between sodium and added sugar and dietary TAC are also needed.

In this study, the significantly decreasing trend in all-cause mortality according to dietary TAC was maintained in model 2 but disappeared after adjusting for smoking status. This could be partly explained by effect of smoking on oxidative stress and the distribution of dietary TAC. Smoking is a well-known lifestyle factor to increase oxidative stress in the body by generating free radicals [8,68]. In addition, although we excluded the outliers of dietary TAC, dietary TAC tended to be right-skewed and a linear trend estimated using the median value of quintiles and quintiles as continuous variables showed different statistical significances. The HRs were lowest in participants with quintile 4 of dietary TAC in all models and the DQIS were consistently highest in quintile 4 of dietary TAC, not in quintile 5. These findings suggest that dietary TAC and mortality may not have a linear relationship. Among various dietary antioxidants, previous studies reported that flavonoids have the highest antioxidant capacities [32] and flavonoids are major contributor to dietary TAC [59]. Recently, a prospective study conducted on 2349 elderly Australians with 14 years of follow-up found that all-cause mortality was the lowest with moderate to high consumption of flavonoids and flavonoid-rich foods [69]. A meta-analysis of prospective studies reported a U-shaped association between vitamin C intake and all-cause mortality [70]. Therefore, further studies are required to investigate an optimal level of dietary TAC with considerations for an individual’s metabolic and inflammation status.

To our knowledge, this is the first study to investigate a relative validity of dietary TAC predicting all-cause mortality in comparison with major DQIS, based on current antioxidant databases and national survey data. On the other hand, this study also has several limitations. First, dietary intake measured only at baseline using a 1-day 24 h dietary recall might not reflect the cumulative usual diet of participants. However, a single day 24 h dietary recall may be adequate to estimate the average intake of a population, if the sample size is large enough [71]. Second, this study did not consider TAC from dietary supplements due to a lack of detailed information on dietary supplements. Third, there might be potential measurement errors due to discrepancies of survey methods between NHANES III and 1999–2006, although we carefully examined and selected dietary variables and other confounding variables to reduce these errors.

## 5. Conclusions

In conclusion, these findings indicate that US adults with high dietary TAC had greater adherence to the DQIS including HEI-2015, AHEI-2010, aMED, and DASH and dietary TAC might be a relatively valid indicator in terms of predicting all-cause mortality in US adults compared to the DQIS. Further studies should expand upon this investigation to assess disease-specific mortality among subpopulations defined by gender, race/ethnicity, and socioeconomic status as well as metabolic and inflammation status.

## Figures and Tables

**Table 1:** Baseline characteristics of study participants according to quintiles of diet quality index scores and dietary total antioxidant capacity (mg VCE/day) ^1^.

	HEI-2015		AHEI-2010		aMED		DASH		Energy-Adjusted TAC	
	Quintile 1 (n = 4759)	Quintile 5 (n = 4759)	Quintile 1 (n = 4759)	Quintile 5 (n = 4759)	Quintile 1 (n = 3028)	Quintile 5 (n = 5057)	Quintile 1 (n = 4556)	Quintile 5 (n = 4660)	Quintile 1 (n = 4759)	Quintile 5 (n = 4759)
Range	12.7–40.3	62.0–99.99	1.8–27.1	47.1–89.5	0–1	5–9	8–17	26–40	0.3–90.5	636.3–6163.1
Gender										
Men	2372 (49.2)	2129 (43.6)	2620 (56.6)	2108 (42.6)	1607 (52.0)	2435 (47.2)	2792 (62.8)	1872 (39.6)	2471 (50.2)	2021 (41.6)
Women	2387 (50.8)	2630 (56.4)	2139 (43.4)	2651 (57.4)	1421 (48.0)	2622 (52.8)	1764 (37.2)	2788 (60.4)	2288 (49.8)	2738 (58.4)
Age (years)										
30–44	2042 (50.1)	1124 (29.4)	2189 (52.3)	1202 (31.6)	1264 (50.0)	1473 (33.6)	2377 (57.9)	1039 (29.4)	1950 (48.3)	1398 (36.4)
45–54	889 (22.2)	751 (21.2)	921 (22.3)	855 (23.9)	576 (22.3)	911 (23.9)	950 (22.9)	688 (20.1)	907 (23.0)	884 (24.4)
55–64	761 (14.2)	893 (19.2)	727 (13.2)	903 (18.2)	489 (13.4)	918 (17.7)	643 (11.9)	879 (18.9)	811 (15.2)	873 (16.8)
65–74	613 (8.2)	1044 (17.8)	526 (7.3)	976 (15.9)	391 (8.5)	952 (15.3)	376 (5.0)	1083 (19.2)	627 (8.4)	824 (13.4)
75–84	359 (4.4)	737 (10.0)	298 (3.9)	653 (8.7)	239 (4.9)	638 (7.9)	168 (1.8)	745 (10.0)	354 (4.1)	597 (7.1)
≥85	95 (0.9)	210 (2.3)	98 (0.9)	170 (1.7)	69 (1.0)	165 (1.6)	42 (0.4)	226 (2.4)	110 (1.1)	183 (2.0)
Race/Ethnicity										
White	2143 (74.3)	2515 (78.1)	2031 (71.1)	2512 (78.9)	1349 (73.8)	2702 (79.3)	1852 (70.7)	2714 (83.3)	2034 (72.9)	2731 (79.9)
Black	1461 (13.4)	819 (7.7)	1549 (14.8)	794 (7.6)	905 (13.3)	935 (8.2)	1582 (15.7)	697 (6.4)	1456 (14.4)	968 (8.5)
Hispanic	1059 (9.0)	1297 (10.1)	1077 (9.9)	1295 (8.5)	713 (9.2)	1258 (8.1)	1006 (9.3)	1141 (7.1)	1171 (9.4)	902 (6.0)
Other	96 (3.3)	128 (4.0)	102 (4.2)	158 (5.0)	61 (3.7)	162 (4.4)	116 (4.2)	108 (3.3)	98 (3.3)	158 (5.5)
Marital status										
Married	2931 (66.4)	3152 (71.4)	2950 (67.1)	3189 (72.3)	1883 (66.8)	3462 (72.7)	2922 (68.4)	3076 (71.5)^2^	2982 (65.9)	3084 (71.2)
Unmarried	1753 (33.6)	1547 (28.6)	1724 (32.9)	1508 (27.7)	1103 (33.2)	1514 (27.3)	1560 (31.6)	1524 (28.5)^2^	1711 (34.1)	1602 (28.8)
PIR										
<1.3	1433 (22.4)	970 (12.4)	1418 (22.6)	953 (11.3)	961 (23.9)	893 (11.1)	1397 (23.9)	916 (11.4)	1486 (22.9)	983 (14.1)
1.3–<1.85	621 (11.0)	520 (8.0)	612 (10.9)	487 (7.5)	425 (11.8)	520 (7.2)	573 (10.4)	467 (7.1)	616 (11.3)	540 (8.0)
≥1.85	2309 (66.6)	2855 (79.7)	2344 (66.5)	2919 (81.2)	1375 (64.2)	3257 (81.8)	2240 (65.7)	2862 (81.5)	2256 (65.7)	2867 (77.8)
Physical activity ^3^										
Sufficient	1612 (37.5)	2291 (54.9)	1634 (38.0)	2285 (54.5)	1001 (37.4)	2480 (56.9)	1510 (36.7)	2310 (57.3)	1583 (37.9)	2041 (47.5)
Insufficient	3147 (62.5)	2466 (45.1)	3125 (62.0)	2473 (45.5)	2027 (62.6)	2576 (43.1)	3046 (63.3)	2348 (42.7)	3176 (62.1)	2718 (52.5)
Smoking ^4^										
Never and former (quit ≥3 years)	2941 (60.3)	4082 (85.5)	2885 (58.7)	3937 (82.6)	1715 (53.1)	4222 (83.5)	2502 (53.4)	4058 (86.5)	2939 (59.6)	3818 (78.5)
Former (quit <3 years)	187 (4.3)	145 (3.4)	194 (4.4)	160 (3.3)	125 (5.2)	173 (3.8)	216 (5.2)	127 (3.4)	181 (4.2)	134 (2.9)
Current	1613 (35.4)	519 (11.1)	1664 (36.9)	644 (14.1)	1180 (41.6)	646 (12.8)	1823 (41.4)	455 (10.1)	1619 (36.2)	789 (18.7)
BMI (kg/m^2^)	28.7 ± 0.2 ^a^	27.2 ± 0.1 ^b^	28.2 ± 0.2 ^a^	27.3 ± 0.1 ^b^	28.4 ± 0.2 ^a^	27.2 ± 0.1 ^b^	28.6 ± 0.2 ^a^	27.0 ± 0.1 ^b^	28.7 ± 0.2 ^a^	27.7 ± 0.1 ^b^
Energy intake (kcal/day)	2082.8 ± 20.2 ^a^	1957.2 ± 18.0 ^b^	2237.7 ± 18.5 ^a^	1978.7 ± 18.0 ^b^	1960.9 ± 25.3 ^a^	2189.3 ± 17.2 ^b^	2425.5 ± 18.8 ^a^	1942.7 ± 16.5 ^b^	2001.6 ± 21.1	1968.7 ± 18.5
History of diabetes	446 (7.1)	772 (12.1)	414 (6.1)	741 (11.8)	319 (7.3)	643 (9.6)^2^	330 (5.7)	721 (11.2)	479 (8.1)	569 (8.7) ^2^
History of CVDs	454 (8.0)	576 (9.3)	416 (7.5)	518 (9.1) ^2^	296 (8.3)	512 (7.9) ^2^	316 (6.3)	575 (9.9)	490 (8.5)	519 (7.9) ^2^
History of hypertension	1539 (29.3)	1806 (34.2)	1498 (28.7)	1722 (32.0)	965 (28.0)	1792 (31.9)	1305 (26.9)	1791 (33.8)	1578 (30.4)	1719 (31.9) ^2^

AHEI-2010, Alternative Healthy Eating Index-2010; aMED, alternate Mediterranean Diet; BMI, body mass index; CVDs, cardiovascular diseases; DASH, Dietary Approaches to Stop Hypertension; HEI-2015, Healthy Eating Index-2015; PIR, poverty-income ratio; TAC, total antioxidant capacity; VCE, vitamin C equivalents. ^1^ All values are presented as means ± SE or n (%) and were statistically different according to quintiles (*p* < 0.05) unless noted otherwise. Log-transformed values were used for statistical tests of continuous variables. Different superscript letters (a,b) indicate significant differences according to quintiles by Tukey-Kramer test (*p* < 0.05). ^2^ Not statistically significant. ^3^ “Sufficient,” performed moderate activity for five or more times per week or vigorous activities per three or more times per week. ^4^ “Never,” never smoked cigarettes or smoked less than 100 cigarettes in an entire lifetime; “former,” smoked at least 100 cigarettes in an entire lifetime but not a current smoker; “current,” smoked at least 100 cigarettes in an entire lifetime and a current smoker.

**Table 2:** Daily food and nutrient intakes and diet quality index scores according to quintile of dietary total antioxidant capacity ^1^.

	Energy-Adjusted Dietary TAC (mg VCE/day)					
	Quintile 1 (n = 4759)	Quintile 2 (n = 4760)	Quintile 3 (n = 4759)	Quintile 4 (n = 4760)	Quintile 5 (n = 4759)	*p* for Trend
Diet quality index scores						
HEI-2015	43.7 ± 0.3 ^a^	50.6 ± 0.3 ^b^	54.7 ± 0.4 ^c^	58.4 ± 0.3 ^d^	54.4 ± 0.4 ^c^	<0.0001
AHEI-2010	32.4 ± 0.4 ^a^	37.2 ± 0.4 ^b^	38.9 ± 0.4 ^c^	40.6 ± 0.3 ^d^	38.5 ± 0.4 ^b,c^	<0.0001
aMED	2.42 ± 0.04 ^a^	3.12 ± 0.04 ^b^	3.60 ± 0.05 ^c^	3.95 ± 0.03 ^d^	3.59 ± 0.04 ^c^	<0.0001
DASH	18.7 ± 0.1 ^a^	21.4 ± 0.1 ^b^	23.1 ± 0.1 ^c^	24.4 ± 0.1 ^d^	22.4 ± 0.1 ^e^	<0.0001
Foods and nutrients						
Total fruits (cup eq.)	0.3 ± 0.03 ^a^	0.7 ± 0.04 ^b^	1.5 ± 0.03 ^c^	2.5 ± 0.04 ^d^	1.7 ± 0.05 ^e^	<0.0001
Whole fruits (cup eq.)	0.3 ± 0.03 ^a^	0.6 ± 0.03 ^b^	1.0 ± 0.03 ^c^	1.5 ± 0.04 ^d^	1.1 ± 0.04 ^e^	<0.0001
Fruit juice (cup eq.)	0.1 ± 0.02 ^a^	0.1 ± 0.01 ^b^	0.5 ± 0.02 ^c^	0.9 ± 0.02 ^d^	0.6 ± 0.03 ^c^	<0.0001
Total vegetables (cup eq.)	1.2 ± 0.05 ^a^	2.0 ± 0.05 ^b^	2.3 ± 0.06 ^b,c^	2.5 ± 0.05 ^b,c^	2.5 ± 0.05 ^c^	<0.0001
Dark green vegetables (cup eq.)	0.0 ± 0.01 ^a^	0.1 ± 0.01 ^b^	0.2 ± 0.01 ^c^	0.3 ± 0.02 ^d^	0.2 ± 0.02 ^c^	<0.0001
Potatoes (cup eq.)	0.5 ± 0.03 ^a^	0.5 ± 0.03 ^a^	0.5 ± 0.03 ^a^	0.4 ± 0.02 ^b^	0.5 ± 0.02 ^a^	0.8258
Whole grains (oz eq.)	0.6 ± 0.04 ^a^	0.7 ± 0.04 ^b^	0.8 ± 0.04 ^b,c^	0.9 ± 0.04 ^d^	0.9 ± 0.04 ^c,d^	<0.0001
Refined grains (oz eq.)	6.2 ± 0.08 ^a^	5.8 ± 0.1 ^a,b^	5.6 ± 0.09 ^b,c^	5.4 ± 0.09 ^c^	5.6 ± 0.09 ^b,c^	0.1752
Dairy (cup eq.)	1.3 ± 0.04 ^a^	1.3 ± 0.03 ^a,b^	1.3 ± 0.04 ^b,c^	1.3 ± 0.04 ^a,b^	1.1 ± 0.04 ^a^	0.4609
Total protein foods (oz eq.)	6.5 ± 0.11	6.5 ± 0.11	6.5 ± 0.11	6.5 ± 0.1	6.6 ± 0.1	0.2915
Red and processed meats (oz eq.)	3.0 ± 0.07 ^a^	2.7 ± 0.07 ^a,b^	2.5 ± 0.07 ^b,d^	2.2 ± 0.07 ^c^	2.5 ± 0.08 ^d^	<0.0001
Seafood (oz eq.)	0.8 ± 0.07 ^a^	0.8 ± 0.07 ^a,b^	0.8 ± 0.07 ^b,c^	1.0 ± 0.09 ^c^	0.9 ± 0.06 ^b,c^	0.0058
Legumes (cup eq.)	0.1 ± 0.01 ^a^	0.2 ± 0.01 ^b^	0.2 ± 0.01 ^b^	0.2 ± 0.01 ^b^	0.2 ± 0.01 ^b^	0.0262
Nuts and seeds (oz eq.)	0.4 ± 0.03 ^a^	0.4 ± 0.03 ^a,b^	0.5 ± 0.05 ^b^	0.5 ± 0.04 ^b^	0.4 ± 0.04 ^b^	0.0206
SSBs (g)^2^	400.9 ± 15.3 ^a^	211.1 ± 11.4 ^b^	152.9 ± 10.0 ^c^	141.5 ± 10.4 ^c^	298.4 ± 12.2 ^d^	0.3658
SFA (% of energy)	11.7 ± 0.12 ^a^	10.8 ± 0.12 ^b^	10.3 ± 0.11 ^c^	9.4 ± 0.1 ^d^	9.8 ± 0.13 ^e^	<0.0001
PUFA (% of energy)	7.3 ± 0.11 ^a^	7.2 ± 0.09 ^a,b^	7.0 ± 0.09 ^b,c^	6.7 ± 0.09 ^c^	7.0 ± 0.1 ^b^	0.1838
MUFA (% of energy)	13.3 ± 0.12 ^a^	12.5 ± 0.12 ^b^	11.9 ± 0.11 ^c^	11.0 ± 0.11 ^d^	11.7 ± 0.13 ^c^	<0.0001
Sodium (mg)	3201.9 ± 33.9 ^a,b^	3279.6 ± 37.4 ^c^	3236.2 ± 38.1 ^a,c^	3166.9 ± 34.3 ^b^	3268.1 ± 31.0 ^a,b,c^	0.3008
Added sugars (teaspoon eq.)	20.2 ± 0.38 ^a^	15.8 ± 0.38 ^b,c^	15.3 ± 0.31 ^b,c^	14.6 ± 0.36 ^b^	16.1 ± 0.31 ^c^	0.9233
Alcohol (drinks)	0.3 ± 0.04 ^a^	0.8 ± 0.05 ^b^	0.6 ± 0.04 ^b^	0.5 ± 0.04 ^b^	0.4 ± 0.04 ^a^	0.0172

AHEI-2010, Alternative Healthy Eating Index-2010; aMED, alternate Mediterranean Diet; DASH, Dietary Approaches to Stop Hypertension; eq., equivalents; HEI-2015, Healthy Eating Index-2015; MUFA, monounsaturated fatty acids; PUFA, polyunsaturated fatty acids, SFA, saturated fatty acids, SSB, sugar sweetened beverage; TAC, total antioxidant capacity; VCE, vitamin C equivalents. ^1^ All values are presented as adjusted means ± SE after being adjusted for age, gender, race/ethnicity, body mass index, and total energy intake. Log-transformed values were used for statistical tests. Different superscript letters (a–e) indicate significant differences according to quintiles by Tukey-Kramer test (*p* < 0.05). ^2^ SSBs included soft drinks, sport and energy drinks, nutritional beverages, and sweetened coffee and tea.

**Table 3:** Multivariable-adjusted hazard ratios and 95% confidence intervals for all-cause mortality according to quintiles of diet quality index scores and dietary total antioxidant capacity (mg VCE/day).

Quintile (Median, Range)	n of Deaths/n	Person-Years	All-Cause Mortality		
			Model 1 ^1^	Model 2 ^2^	Model 3 ^3^
HEI-2015					
Q1 (34.5, 12.7–40.3)	1507/4759	72,190.7	1.00	1.00	1.00
Q2 (43.9, 39.6–47.9)	1474/4760	72,415.5	0.81 (0.74–0.90)	0.82 (0.74–0.91)	0.87 (0.78–0.97)
Q3 (50.9, 47.0–54.9)	1596/4759	70,543.8	0.82 (0.73–0.91)	0.85 (0.76–0.95)	0.92 (0.82–1.03)
Q4 (58.1, 53.9–63.8)	1698/4760	70,431.9	0.80 (0.72–0.90)	0.85 (0.76–0.97)	0.93 (0.82–1.06)
Q5 (69.3, 62.0–99.99)	1831/4759	68,750.0	0.70 (0.63–0.78)	0.76 (0.68–0.85)	0.87 (0.77–0.98)
*p* for trend ^4^			<0.0001	0.0002	0.1178
*p* for trend ^5^			<0.0001	0.0005	0.1529
AHEI-2010					
Q1 (21.0, 1.8–27.1)	1436/4759	73,429.5	1.00	1.00	1.00
Q2 (29.7, 24.9–34.5)	1589/4762	71,136.1	0.86 (0.78–0.94)	0.86 (0.77–0.95)	0.90 (0.81–1.01)
Q3 (36.5, 31.9–41.5)	1665/4757	70,485.7	0.79 (0.71–0.89)	0.81 (0.72–0.90)	0.88 (0.79–0.97)
Q4 (43.9, 38.7–49.6)	1727/4760	68,953.6	0.81 (0.72–0.90)	0.81 (0.73–0.91)	0.89 (0.80–1.00)
Q5 (54.5, 47.1–89.5)	1689/4759	70,327.0	0.71 (0.63–0.80)	0.76 (0.67–0.86)	0.84 (0.74–0.94)
*p* for trend ^4^			<0.0001	<0.0001	0.0082
*p* for trend ^5^			<0.0001	<0.0001	0.0085
aMED					
Q1 (1, 0–1)	1055/3028	45,844.3	1.00	1.00	1.00
Q2 (2, 2–2)	1652/4658	68,945.8	0.87 (0.77–0.99)	0.88 (0.76–1.01)	0.92 (0.79–1.06)
Q3 (3, 3–3)	2074/5947	87,805.8	0.77 (0.68–0.87)	0.79 (0.69–0.91)	0.86 (0.75–0.99)
Q4 (4, 4–4)	1713/5107	75,947.4	0.66 (0.58–0.76)	0.68 (0.59–0.79)	0.78 (0.67–0.89)
Q5 (5, 5–9)	1612/5057	75,788.6	0.63 (0.55–0.73)	0.68 (0.59–0.79)	0.79 (0.69–0.90)
*p* for trend ^4^			<0.0001	<0.0001	<0.0001
DASH					
Q1 (15, 8–17)	1186/4556	72,214.5	1.00	1.00	1.00
Q2 (19, 18–20)	1620/5048	76,403.8	0.79 (0.71–0.89)	0.81 (0.73–0.90)	0.87 (0.78–0.96)
Q3 (22, 21–23)	1495/4689	66,929.1	0.65 (0.57–0.73)	0.67 (0.59–0.76)	0.75 (0.66–0.86)
Q4 (24, 23–26)	1899/4844	70,141.8	0.66 (0.58–0.75)	0.70 (0.62–0.80)	0.81 (0.71–0.92)
Q5 (28, 26–40)	1906/4660	68,642.6	0.61 (0.54–0.70)	0.68 (0.59–0.77)	0.80 (0.70–0.92)
*p* for trend ^4^			<0.0001	<0.0001	0.0074
*p* for trend ^5^			<0.0001	<0.0001	0.0123
Energy-adjusted TAC					
Q1 (50.5, 0.3–90.5)	1545/4759	71,411.7	1.00	1.00	1.00
Q2 (122.3, 81.0–171.3)	1555/4760	71,676.4	0.86 (0.79–0.94)	0.87 (0.79–0.95)	0.91 (0.83–0.999)
Q3 (221.4, 164.4–296.6)	1673/4759	70,364.3	0.76 (0.70–0.84)	0.80 (0.73–0.88)	0.88 (0.80–0.97)
Q4 (403.8, 292.2–645.3)	1681/4760	70,331.8	0.71 (0.64–0.78)	0.76 (0.69–0.84)	0.85 (0.78–0.94)
Q5 (1201.4, 636.3–6163.1)	1652/4759	70,547.7	0.75 (0.68–0.82)	0.79 (0.71–0.88)	0.88 (0.79–0.98)
*p* for trend ^4^			0.0007	0.0132	0.2553
*p* for trend ^5^			<0.0001	<0.0001	0.0293

AHEI-2010, Alternative Healthy Eating Index-2010; aMED, alternate Mediterranean Diet; DASH, Dietary Approaches to Stop Hypertension; HEI-2015, Healthy Eating Index-2015; TAC, total antioxidant capacity; VCE, vitamin C equivalents. ^1^ Adjusted for age, gender, race/ethnicity, body mass index, and total energy intake. ^2^ Additionally adjusted for poverty-income ratio, marital status, physical activity, history of cardiovascular diseases, history of diabetes, and history of hypertension. ^3^ Additionally adjusted for smoking status. ^4^ *p* for trend was estimated using the median value of each quintile. ^5^ *p* for trend was estimated using quintiles as continuous variables.

## Supplementary Materials

The following are available online at https://www.mdpi.com/2072-6643/12/5/1210/s1, Table S1: Baseline characteristics of study participants in NHANES III and 1999–2006.
