# Supplementary material for: Relative Validity of Dietary Total Antioxidant Capacity for Predicting All-Cause Mortality in Comparison to Diet Quality Indexes in US Adults
Source: Nutrients. 2020 Apr 25;12(5):1210. doi: 10.3390/nu12051210 (PMC7282024; doi:10.3390/nu12051210)
Supplement: Supplementary file 1 [file nutrients-12-01210-s001.pdf]

Table S1. Baseline characteristics of study participants in NHANES III and 1999–2006[1].

	NHANES III (n=11375)	NHANES 1999–2006 (n=12422)
Gender		
Men	5383 (47.5)	6236 (48.7)
Women	5992 (52.5)	6186 (51.3)
Age (years)		
30–44	4313 (45.0)	3921 (38.2)
45–54	1789 (18.7)	2508 (25.3)
55–64	1866 (15.4)	2152 (16.1)
65–74	1860 (13.0)	2034 (11.9)
75–84	1217 (6.4)	1366 (6.7)
≥85	330 (1.4)	441 (1.8)
Race/Ethnicity		
White	4984 (77.8)	6257 (73.6)
Black	3110 (10.6)	2556 (11.0)
Hispanic	3122 (8.5)	3194 (10.9)
Other	159 (3.1)	415 (4.5)
Marital status		
Married	7468 (71.5)	7850 (68.4)
Unmarried	3883 (28.5)	4227 (31.6)
PIR		
<1.3	3172 (16.2)	2987 (17.9)
1.3–<1.85	1447 (10.3)	1472 (9.3)
≥1.85	5708 (73.5)	7017 (72.7)
Physical activity[2]		
Sufficient	4062 (41.1)	5360 (48.3)
Insufficient	7312 (58.9)	7060 (51.7)
Smoking[3]		
Never and former (quit ≥3 years)	8042 (69.3)	9356 (73.9)
Former (quit <3 years)	455 (4.7)	417 (3.5)
Current	2827 (26.0)	2618 (22.5)
BMI (kg/m^2)	27.0 ± 0.1	28.5 ± 0.1
Energy intake (kcal/day)	2048.4 ± 16.1	2101.8 ± 13.5
Dietary TAC (mg VCE/day)	475.8 ± 11.8	455.7 ± 9.8
History of diabetes	1144 (6.6)	1729 (10.2)
History of CVDs	823 (4.5)	1676 (10.5)
History of hypertension	3654 (28.0)	4578 (32.9)

BMI, body mass index; CVDs, cardiovascular diseases; NHANES, National Health and Nutrition Examination Survey; PIR, poverty-income ratio; TAC, total antioxidant capacity; VCE, vitamin C equivalents. [1] All values are presented as means ± SE or n (%). [2] "Sufficient," performed moderate activity for five or more times per week or vigorous activities per three or more times per week. [3] "Never," never smoked cigarettes or smoked less than 100 cigarettes in an entire lifetime; "former," smoked at least 100 cigarettes in an entire lifetime but not a current smoker; "current," smoked at least 100 cigarettes in an entire lifetime and a current smoker.
